# Application of the adverse outcome pathway (AOP) concept to structure the available in vivo and in vitro mechanistic data for allergic sensitization to food proteins

**DOI:** 10.1186/s13601-017-0152-0

**Published:** 2017-05-12

**Authors:** Jolanda H. M. van Bilsen, Edyta Sienkiewicz-Szłapka, Daniel Lozano-Ojalvo, Linette E. M. Willemsen, Celia M. Antunes, Elena Molina, Joost J. Smit, Barbara Wróblewska, Harry J. Wichers, Edward F. Knol, Gregory S. Ladics, Raymond H. H. Pieters, Sandra Denery-Papini, Yvonne M. Vissers, Simona L. Bavaro, Colette Larré, Kitty C. M. Verhoeckx, Erwin L. Roggen

**Affiliations:** 1 TNO, PO Box 360, 3700 AJ Zeist, The Netherlands; 2 University of Warmia and Mazury, Olsztyn, Poland; 3 Instituto de Investigación en Ciencias de la Alimentación, Madrid, Spain; 4 Utrecht University, Utrecht, The Netherlands; 5 University of Evora, Évora, Portugal; 6 Institute of Animal Reproduction and Food Research of Polish Academy of Sciences, Olsztyn, Poland; 7 Wageningen University and Research, Wageningen, The Netherlands; 8 University Medical Center Utrecht, Utrecht, The Netherlands; 9 DuPont Company, Newark, NJ USA; 10 INRA, Nantes, France; 11 Nestlé Ltd., Nestlé Research Center, Lausanne, Switzerland; 12 Institute of Sciences of Food Production, National Research Council, Bari, Italy; 13 3Rs Managing and Consulting ApS, Lyngby, Denmark

**Keywords:** Adverse outcome pathway, Key events, Key event relations, Food allergy, Sensitization, Mechanistic understanding, Food proteins, Molecular initiating event

## Abstract

**Background:**

The introduction of whole new foods in a population may lead to sensitization and food allergy. This constitutes a potential public health problem and a challenge to risk assessors and managers as the existing understanding of the pathophysiological processes and the currently available biological tools for prediction of the risk for food allergy development and the severity of the reaction are not sufficient. There is a substantial body of in vivo and in vitro data describing molecular and cellular events potentially involved in food sensitization. However, these events have not been organized in a sequence of related events that is plausible to result in sensitization, and useful to challenge current hypotheses. The aim of this manuscript was to collect and structure the current mechanistic understanding of sensitization induction to food proteins by applying the concept of adverse outcome pathway (AOP).

**Main body:**

The proposed AOP for food sensitization is based on information on molecular and cellular mechanisms and pathways evidenced to be involved in sensitization by food and food proteins and uses the AOPs for chemical skin sensitization and respiratory sensitization induction as templates. Available mechanistic data on protein respiratory sensitization were included to fill out gaps in the understanding of how proteins may affect cells, cell–cell interactions and tissue homeostasis. Analysis revealed several key events (KE) and biomarkers that may have potential use in testing and assessment of proteins for their sensitizing potential.

**Conclusion:**

The application of the AOP concept to structure mechanistic in vivo and in vitro knowledge has made it possible to identify a number of methods, each addressing a specific KE, that provide information about the food allergenic potential of new proteins. When applied in the context of an integrated strategy these methods may reduce, if not replace, current animal testing approaches. The proposed AOP will be shared at the www.aopwiki.org platform to expand the mechanistic data, improve the confidence in each of the proposed KE and key event relations (KERs), and allow for the identification of new, or refinement of established KE and KERs.

## Background

Consumers are exposed to increasing numbers of novel proteins or protein-containing products (e.g. insect burgers or proteins derived from bacteria grown on waste streams). These sustainable protein-rich food products are to solve the food insecurity problem but require a comprehensive risk assessment complying with the European ‘Novel Food’ law. Additional knowledge and biological tools are needed to support the prediction of the risk for food allergy development and the potential severity of the reaction [1, 2]. This constitutes a major public health problem and a challenge to risk assessors and managers [3, 4].

Like other allergies, food allergy has a non-symptomatic sensitization phase and a symptomatic elicitation phase. Food-associated adverse reactions can be immunoglobulin E (IgE) mediated, non-IgE mediated or both [5]. This paper focusses on the current understanding of the cellular and molecular mechanisms driving sensitization induction resulting in IgE-mediated allergy.

The mode of action (MOA) of sensitization and IgE mediated allergy to food proteins in predisposed individuals is poorly understood [6]. It is recognized that food processing, oral uptake and digestion affect the characteristics of food and food proteins [7]. Thus, acquiring a good understanding of the MOA requires well-characterized food and food protein samples for in vivo challenges in animals or preferentially humans. Such samples are now made available by the INFOGEST Cost Action [8].

There is a substantial body of in vivo and in vitro data describing molecular and cellular events potentially involved in food sensitization. However, these events have not been organized in a sequence of related events that is plausible to result in sensitization, and useful to challenge current hypotheses [9, 10]. The aim of this paper is to collect and structure the current mechanistic understanding of sensitization induction to food proteins by applying the concept of adverse outcome pathway (AOP).

## Main text

### AOPs for food sensitization: a proposal

The AOP concept is a framework for collecting and organizing information relevant to an adverse outcome at different levels of biological organization. It is believed that AOPs based on available information on substance–response and response–response relationships allow the development of relevant predictive animal-free test methods and approaches, as well as the contextualization of the results across a diverse range of biological mechanisms and toxicity endpoints. The Organisation for Economic Cooperation and Development (OECD) provides an international hub for constructing, reviewing, and using AOPs with the help of a suite of tools comprising the AOP knowledge base, including the AOP Wiki (www.aopwiki.org). This AOP Wiki provides a collaborative platform for constructing AOPs and can be used by groups that have a proposal for an AOP [11].

**Fig. 1:**
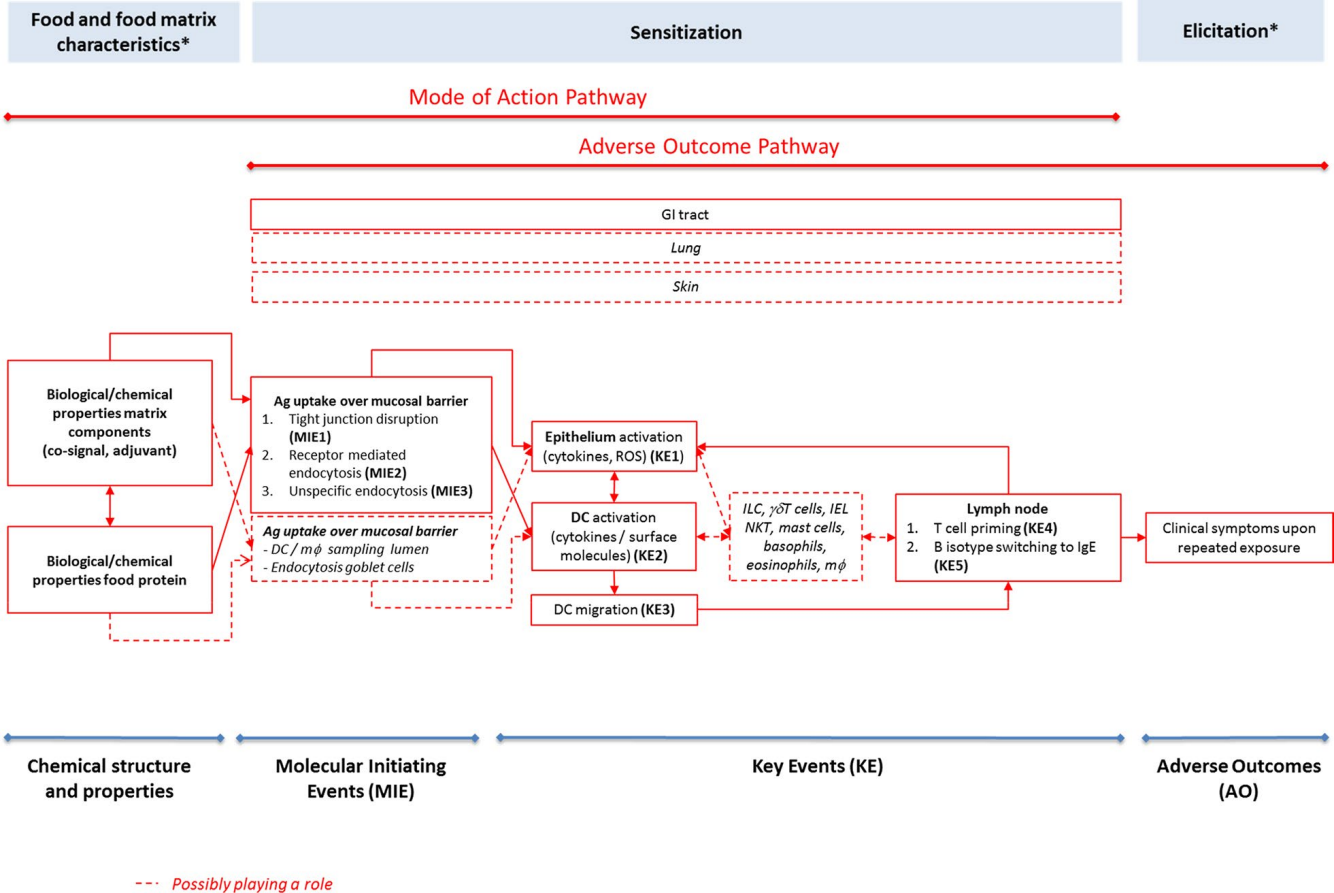
A tentative MOA including an AOP describing the mechanistic events driving food sensitization induction. *Solid boxes* and *arrows* represent events and relationships with substantial evidence for a role in sensitization induction to food proteins. *Dashed boxes* and *dashed arrows* represent events, organs cellular components or relationships with circumstantial evidence for a role in the AOP. *Ag* antigen, *GI* gastro-intestinal, *ILC* innate lymphoid cells, *mϕ* macrophages, *NKT* natural killer cells, *IEL* intraepithelial lymphocytes. *Outside the scope of this manuscript

This manuscript proposes an AOP for food sensitization (Fig. 1). The information on mechanisms and pathways evidenced to be involved in sensitization by food and food proteins is structured using the AOP for chemical skin sensitization [12] and respiratory sensitization [13] induction as templates. Available mechanistic data on protein respiratory sensitization [14] were considered to fill out gaps in the understanding of how proteins may affect cells, cell–cell interactions and tissue homeostasis. Figure 1 depicts a proposal for the AOP of food sensitization including all key events and cellular players with substantial (solid lines) or circumstantial (dashed lines) evidence for a role in the sensitization induction to food proteins. In this review, the successive sections describe events following the common structure of an AOP consisting of a molecular initiating events (MIE) and a series of key events (KEs) that eventually lead to the occurrence of clinical symptoms upon repeated exposure in the elicitation phase.

### Molecular processes in the AOP for food sensitization

#### Bioavailability: protein and protein fragments acquire access to the relevant immune cells

##### Acquiring access to the underlying immune system via the gastrointestinal tract

**Fig. 2:**
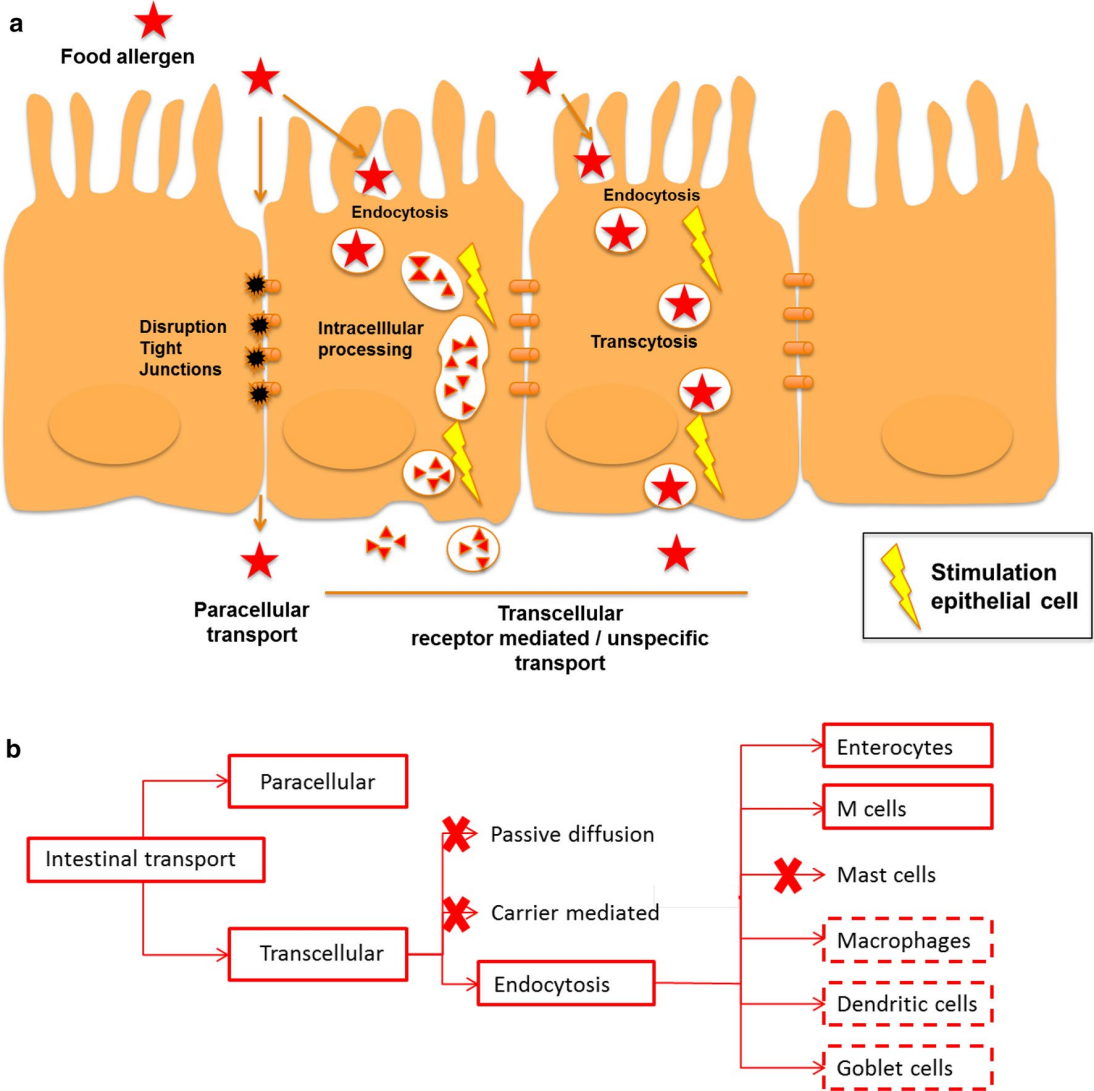
Molecular initiating events (MIE) that initiate food sensitization. **a** Food allergen interaction with mucosal surfaces of the gut intestine may result in disruption of tight junctions, receptor mediated or unspecific transcellular transport of food allergens across the gut epithelium. These events can be the initiating events that results in the activation of epithelial and innate cells such as dendritic cells.** b** Mechanisms and cells involved in intestinal protein transport in non-sensitized individuals. *Solid boxes* represent events with substantial evidence of being involved in food protein transport. *Dashed boxes* represent events that are possibly involved in food protein transport. *Crosses* indicate events and cell types of which there is no indication that they play a role in the transport of food proteins across the gut barrier in non-sensitized individuals

The multiple facets of intestinal permeability and epithelial handling of dietary antigens were previously reviewed [15]. The following sections intend to capture the current understanding of the role of transport in sensitization induction as schematically outlined in Fig. 2.

Impairment of tight junctions may be implicated in sensitization induction. Paracellular transport is mainly determined by pore size in tight junction and only concerns molecules of MW < 600 Da [16, 17]. Proteins that are transported via this paracellular route are not exposed to lysosomes in the enterocyte and therefore are not degraded [18, 19]. Passage of intact protein may allow sensitization of immune cells in the subepithelial compartment (Fig. 2a).

Several in vitro studies suggest that gluten (gliadin), kiwi (Act d 1) and peanut (Ara h 2) allergens facilitate absorption by modulating tight junctions [20–23]. Act d 1, in analogy with house dust mite (HDM) Der p 1 [24], may cause a protease-dependent disruption of the tight junctions of the epithelial cell (EC) layer (Fig. 2a) For Ara h 2 the effect on the tight junctions and barrier integrity appears to involve endocytosis and downregulation of the zink finger protein A20, a crucial gatekeeper preserving tissue homeostasis with ubiquitin-regulatory activities [21].

Among various intestinal innate immune cells, mast cells (MCs) play critical roles in maintaining gut immune homeostasis. Studies on sensitized individuals revealed that the integrity of the tight junctions is decreased by mast cell-derived mediators like tryptase and IL-4 [25–30]. In murine models, the importance of MCs for the development of food allergies has also been clearly shown. Mice presensitized with alum and OVA develop allergic diarrhoea after oral OVA inoculation [31]. Inhibitors of serotonin and histamine as well as depletion of MCs by an anti-c-kit monoclonal antibody reduce the occurrence of allergic diarrhoea. Therefore, MCs as well as serotonin and histamine derived from them are involved in the allergic diarrhoea in this model [31]. These murine and human data clearly show the importance of MCs in the gut homeostasis and the effect of MC activity by allergens resulting in inflammatory responses in mucosal compartments [32]. However, in the context of food allergy, the importance of MCs has only been described in already sensitized animals/individuals. To date there is no data available supporting the role of mast cells in sensitization induction to food proteins as depicted in Fig. 2b.

 Transcellular transport mechanisms provide intact protein with free passage to the sub-epithelial compartment. Transcellular transport of proteins and peptides can occur via different mechanisms as illustrated in Fig. 2a, b. Most dietary proteins or peptides are absorbed actively by ECs in non-sensitised persons by endocytosis at the apical membrane and undergo transcytosis toward the lamina propria (LP). An estimate of 70–90% of the transported protein is degraded by intracellular enzymes into amino acids and peptides [33, 34].

In vitro studies showed that intact soybean allergen P34 and bovine β-lactoglobulin cross the epithelial barrier by transcytosis, while tryptic fragments of β-lactoglobulin followed para- and transcellular routes [35, 36]. Thus, protein resisting proteolysis in the gastrointestinal tract is transported intact through the intestinal epithelial barrier, and may enhance the risk for sensitisation induction.

In sensitized persons, T_h_2 or innate lymphoid cells (ILC)-2-derived IL-13 with other inflammatory mediators (e.g. IFN-γ, TNF-α and IL1-β) stimulate transport via the CD23 receptor, which is overexpressed by enterocytes in sensitised persons. Antigen-IgE complexes are transcellularly transported by the CD23 receptor [18]. A role for CD23-mediated transport in sensitization induction must still be documented.

In other studies, receptor-mediated transcellular transport via the CD23 receptor appears to protect proteins from lysosomal degradation [18, 19], however as explained above, CD23-mediated transport has only been described in already sensitized individuals.

M cells transport protein directly to the underlying immune cells. M cells are specialized ECs covering the Peyer’s, caecal and colonic patches. These cells deliver intact proteins and particles directly to the gut associated lymphoid tissues (GALT), and therefore may play an important role in the regulation of immunological reactions to dietary antigens [37, 38].

The current perception is that particulate or aggregated antigens are taken up by M cells, resulting in antigen-specific local or systemic immune responses [39, 40]. This perception is supported by a study in mice showing that pasteurization shifted the transport of soluble β-lactoglobulin and α-lactalbumin from transcytosis through enterocytes to transport of protein aggregates by M cells to the Peyer’s patches, and higher IgE and cytokine (IL-5, IL-13, IFN-γ, IL-10) levels [41]. However, another study demonstrating the presence of soluble protein as well as protein bodies of digested peanut in the cytoplasm of M cells within intestinal Peyer’s patches in BALB/c mice [42] suggests that additional factors may contribute to the induction of adverse responses.

Monocytes and DCs sample protein directly from in the gut lumen. Both cells can sample the gut lumen using their pseudopodia. Tight junctions are opened and after sampling new tight junctional complexes between adjacent ECs are formed. Luminal sampling is upregulated in inflammatory conditions [43, 44]. However, information is lacking whether protein transport occurs via these cells.

The role of goblet cells in protein up-take and sensitization induction seems limited. In healthy human ileum and jejunum goblet cells can transport small peptides (10 kDa) and particles across the intestinal wall [45]. A role for these cells in uptake of food proteins has not yet been documented, nor their role in sensitization induction to food proteins.

##### Acquiring access to the underlying immune system after cutaneous and respiratory exposure

There is growing evidence ascribing food proteins the capacity to induce sensitization when contacting the skin, and potentially the respiratory tract. How these proteins acquire access to the cutaneous immune cells that drive sensitization of the gastrointestinal tract is not sufficiently understood yet. In contrast, the mechanistic understanding of protein-induced sensitization of the respiratory tract is growing [14].

Evidence for gastrointestinal tract sensitization following cutaneous exposure exists. Mice exposed via different routes (intragastrically, cutaneously, intranasally or sublingually) to α-lactalbumin in the presence of cholera toxin were all sensitized, however sensitization via the skin resulted in the highest IgE levels [46]. Supporting evidence emerged from studies using hazelnut protein [47] and ovalbumin [48] as model proteins. In each case, oral as well as intragastric challenge led to anaphylactic symptoms after skin sensitization.

Even though human skin appears to be less penetrable than murine skin, epidemiological studies in human populations seem to confirm the skin as a relevant route for food sensitization induction and allergy in man.

High cutaneous exposure to peanut increases the risk of peanut allergy in human [49]. The use of peanut-protein containing skin creams was found to correlate with peanut allergy in children. These creams were mostly used to relieve eczema, i.e. damaged skin with increased permeability for e.g. proteins [50]. The importance of the quality of the skin barrier was demonstrated by the impact of filaggrin mutation/expression on human skin integrity and permeability, and its correlation with increased incidence of sensitization [51]. In Japan, at least 1800 individuals were sensitized following application of a facial soap containing hydrolysed wheat proteins (HWP). An epidemiological relationship was documented between wheat allergy and contact exposure to HWP in Japanese women [52]. Cutaneous sensitization to latex correlates with allergic symptoms upon ingestion of cross-reacting foods e.g. avocado, banana and kiwi. However, it cannot be excluded that oral exposure to these foods may lead to allergic reactions to cutaneous latex exposure [53].

Against the evidence in favor of the skin being a route for sensitization to food and food proteins stand data from studies in mice and humans showing that desensitization can be achieved to food and aero-allergens by applying antigen cutaneously [46].

Evidence for gastrointestinal tract sensitization following respiratory exposureis more ambiguous. The contribution of the respiratory tract to sensitization induction of the gastrointestinal tract is less clear, despite a growing understanding of how proteins may acquire access to immune cells and trigger inflammation [14].

Dunkin et al. [46] presented data suggesting that mice are sensitized by exposure of the respiratory tract to α-lactalbumin in the presence of cholera toxin. However, respiratory sensitization was less effective than cutaneous sensitization as judged from the IgE levels induced by subsequent oral challenges.

In humans, the second most significant risk factor associated with life-threatening asthma is a history of an asthma attack precipitated by food. A clear link between the pathophysiology of food and respiratory allergy is not yet established, but two hypotheses are currently pursued: (i) effector cells resulting from gastral sensitization populate the respiratory tract; (ii) chronic inhalation of food particles results in direct sensitization of the respiratory tract [54]. These hypotheses are not mutually exclusive. The oral allergy syndrome is currently believed to result from respiratory sensitization to pollen (e.g. Bet v 1 protein) in conjunction with oral exposure to apple or carrot, containing cross-reactive Bet v 1 homologues. However, it cannot be excluded that oral ingestion of apple or carrot, results in sensitization and subsequent cross-reactivity to inhaled pollen [55]. There is also evidence suggesting that respiratory sensitization to food proteins not necessarily develops into food allergy. Indeed, to it has been reported that bakers with occupational asthma following exposure to wheat flower can safely consume wheat products [56].

#### The molecular initiation event (MIE): lessons learned from respiratory sensitization

The innate immune system is an evolutionary conserved system equipped with a range of pattern recognition receptors (PRRs) with specificity for pathogen-associated molecular patterns (PAMPs) on microorganisms, parasites and fungi, and danger-associated molecular patterns (DAMPs). These interactions trigger an inflammatory response driving an adaptive immune response [57].

The molecular basis for the propensity of specific proteins to induce sensitization and subsequent allergic responses is still poorly defined. Studies addressing sensitization of the respiratory tract revealed that the ability of proteins to trigger sensitization and allergy is a function of their ability to interact with pathways of innate immune recognition and activation at mucosal surfaces. Potential MIEs relate to proteolytic activity, engagement of PRRs, molecular mimicry of Toll-like receptor (TLR) signalling complex molecules, lipid binding activity, and oxidant potential [14].

The relevance of these pathways for food sensitization induction in general remains to be fully established. The current understanding of the mechanisms applied by food and food proteins (e.g. gluten, kiwi, peanut allergens) suggest that interaction with tight junctions may represent a MIE (MIE-1). Receptor-mediated transcellular transport via e.g. the CD23 receptor may be an alternative route (MIE-2) although its relevance for sensitization induction is not fully established. Endocytosis by either ECs or M cells may be a less well defined MIE (MIE-3) resulting in regulation of gene expression and disturbance of tissue homeostasis or induction of inflammation by direct contact of the potential allergen (e.g. peanut allergens) with antigen presenting cells (APC)s.

Animal experimentation has clearly presented evidence that matrix components are relevant in the sensitisation process. For instance, Van Wijk et al. [58] showed that, in the popliteal lymph node assay in C3H/HeOuJ-mice, purified peanut allergens were unable to induce immune activation, opposed to immunisation with whole peanut extract. In line with this, Wavrin et al. [59] demonstrated a much higher Th2-like response to whole milk in BALB/cJ mice, than to purified β-lactoglobulin. Matrix-derived (poly-)saccharides are involved, via heat-induced glycation, in sensitisation for peanut allergen in BALB/c-mice [60]. Heat-induced glycation of ovalbumin led to the induction of a Th2-skewing milieu in a human DC/T cell co-culture model, in which a role for the mannose receptor is suggested [61].

Similarly, c-type lectins receptors (CLRs) have been highlighted as important factors in initiating and modulation allergic responses [62]. For example, Dectin-1 recognizes β-glucans, a carbohydrate present in cells walls of many, if not all fungal species, and thereby promotes lung immunopathology during fungal allergy [63]. Dectin-1 has also been shown to limit gut mucosal inflammation induced by fungi [64]. In HDM allergy, Dectin-2 can recognize HDM and induce the release of cysteinyl leukotriene, which is, as well as IL-33, essential for the initiation of airway inflammation and promotion of subsequent Th2 immunity in response to HDM [65, 66]. In murine models, Dectin-2 is involved in the development of HDM-allergy during both the sensitization and challenge stages [66, 67]. Likewise, the mannose receptor has been shown to act on human DCs as receptor for HDM (Der p 1 and Der p 2), cockroach (Bla g 2), dog (Can f 1) and peanut (Ara h 1) and shown in mice to play a crucial role in Th2 cell polarization (reviewed by [62]). Several DC-SIGN—binding glycoproteins were identified in common allergenic foods at the difference of food that less frequently induce allergy [68]. Also lipids are claimed to be involved in the sensitization process [69]. Recently, an adjuvant role for the peanut allergen Ara h 1 in sensitisation for Ara h 6 was suggested [70].

Details on the molecular mechanisms of the adjuvant effects that can be exerted by matrix components are mostly still lacking, but this does not preclude their relevance and importance. Additional knowledge into the identification of such matrix components is required and will help to develop tools to incorporate such adjuvancy into model systems. Specific PRRs, such as the mannose receptor, appear to be involved and (co-)activation of e.g. other CLRs, TLRs or G-protein coupled receptors may turn out to be involved.

#### Key events (KE): cellular ‘innate’ events at epithelial (KE 1) and DC (KE 2 and 3) level—setting the scene for inflammation

The intestinal epithelium is a cell monolayer (with diverse cells: enterocytes, Goblet cells, Paneth cells, endocrine cells, M cells) between the lumen and the immune compartment with inductive Peyer’s and colonic patches, draining mesenteric lymph nodes (MLN) and the scattered immune cells in the LP [71]. The role of intestinal epithelium in allergic sensitization induction is recognized but the mechanistic understanding of the processes involved is limited when compared to skin and respiratory sensitization.

##### KE 1: Sensitizer-related inflammatory responses at epithelial level

It is widely accepted that sensitization involves factors from gut epithelium which are activated during cellular stress. Such factors include radical oxygen species (ROS), Th2 driving mediators (e.g. IL-1, IL-18, IL-25, IL-33, TSLP) and mucus. There is limited availability of mechanistic data on pathways driving the allergic sensitization via the intestinal mucosa, however, much can be learned from pathways involved in skin and lung sensitization.

The role of ROS in allergen-induced skin sensitization was reviewed [72]. Evidence for a role of ROS in respiratory sensitization induction is provided by allergens with proteolytic activity (e.g. HDM allergen Der p 1). There is evidence that cysteine proteases may induce stress and elevated ROS levels e.g. through cleavage and activation of the Protease-Activated Receptor (PAR) 2 [24], and inactivation of lung surfactant proteins (e.g. SP-A, SP-D). Consequently, tight junction proteins (e.g. ZO-1 and occluding) become accessible for protease activity, leading to increased transepithelial access of allergens to immune cells [73, 74]. If and how this information can be translated to gastrointestinal tract is not yet clear, but it is clear that its epithelial lining is used to deal with protease activity involved in digestion.

ROS activation is also implicated in the sensitization to pollen allergens exerting [NAD(P)H] oxidase activity, an enzyme activity also found in mitochondria and driving intracellular ROS production [75]. Endocytosis of this pollen-derived enzyme increases ROS production and results in degradation of endogenous hyaluronic acid (HA), and TLR2 and TLR4 activation. This may be of relevance as a variety of studies implicate MyD88-mediated TLR2 and TLR4 signalling in the induction of T_h_2 immune responses leading to allergic respiratory inflammation and promotion of T_h_17 responses [14, 76].

ROS production may be induced directly via TLR activation. The HDM allergen Der p 2 reveals sequential homology to the MD-2-related lipid-recognition (ML) domain family, which is a well-characterized member of the TLR4 signalling complex. There is compelling evidence demonstrating that Der p 2 exhibits the same function as ML. Thus, the intrinsic adjuvant activity of MD-2 homologous allergens and their lipid cargo is likely to have wide generality as a mechanism underlying sensitization induction [77].

Activation of TLR requires phosphorylation by c-Src signals and activation of phosphoinositide 3-kinase and phospholipase C γ for activating NF-κB and chemokine expression leading to lymphocyte recruitment to the lung and increase in mucus production. Subsequently, Ca^2+^-dependent proteases cleave the transmembrane proteins occludin and e-cadherin on ECs promoting transmigration of leukocytes [78]. NFкB activation is causally related to increased release by ECs of IL-33, IL-25 and TSLP, endogenous danger factors (e.g. high-mobility group box-1 (HMGB-1), uric acid and ATP, DC activation and migration, and the induction of ovalbumin and Der p 2 sensitization [79]. The release of ATP and uric acid drives the activation of the NLRP3 inflammasome complex resulting in cleavage of pro-IL-1β to mature IL-1β through caspase 1. IL-1β creates a pro-inflammatory micro-environment with the production of IL-6 and chemokines that mobilize neutrophils and enhance T_h_17 cell differentiation [80]. Uric acid may play an important role in T_h_2 skewing [81].

The limited mechanistic data suggest that similar TLR-dependent and -independent pathways may drive allergic sensitization via the intestinal mucosa. Epithelium-derived factors (IL-33, TSLP, IL-25) identified in studies focusing on HDM-induced sensitization were observed during\ intestinal peanut sensitization in mice, of which it was shown that IL-33 is important for OX40L expression on DC and the development of peanut allergy (see KE 2) [82]. Furthermore, α-amylase inhibitor from cereals activates TLR4 mediated processes [83]. Kong et al. demonstrated that uric acid is a critical signal for the induction of peanut allergy [84].

##### KE 2: Sensitizer-related inflammatory responses at DC level

PRR, TLR and ROS signalling pathways are also active in endothelial cells, macrophages, fibroblasts as well as DCs. These cell types may therefore contribute to the induction of inflammation and sensitization by employing the same mechanisms as described for ECs.

In vitro data revealed that the allergen Pru p 3, but not the non-allergenic LPT 1 variant, crosses the epithelial barrier and induces production of the T_h_2 skewing cytokines TSLP, IL-25, and IL-33. In addition, Pru p 3, but not LPT 1, triggered the expression of inflammatory cytokines such as IL1β, IL6, and IL10 in a co-culture of Caco-2 cells and human peripheral blood mononuclear cells. The highest induction was observed for IL1β, which is related to a T_h_2 response and antibody production [85, 86].

The importance of EC-derived IL-33 for in vivo DC activation was demonstrated using a murine model for intragastric induced peanut allergy. It was observed that peanut administered in the presence of cholera toxin increased the expression of OX40L, a co-signaling molecule required for proper T cell activation [82]. In IL-33 receptor knock out mice this increase was not observed. TSLP and IL-25 protein levels were increased in duodenal tissue of peanut allergic mice, but TSLP or IL-25 receptor (IL-17-RB) knock out (KO) mice revealed no driving role for IL-25 or TSLP in peanut sensitization. These data show that IL-33 is sufficient for the observed increase in OX40L expression, expansion of ILCs and the development of peanut allergy [82].

DCs, among other cells, can sample food protein directly from the gut lumen using pseudopodia. Alternatively, they can come in direct contact with intact protein after paracellular or transcellular transport through the ECs, or by M cell mediated transport. Driven by microenvironment changes and contact with the protein, DCs upregulate the expression of MHC II, costimulatory molecules such as CD54, CD80, and CD86, and receptors that are essential for migration (e.g. CCR7) [87].

While proteins are recognized to drive the activation of DCs in an inflammatory context, the observed suppression of LPS-induced IL-12 production suggest that HDM and peanut allergens may be capable of suppressing T_h_1 driving DC responses [82].

Eosinophils constitute about 20% of the leukocytes in the LP and appear to be required for the induction of CCR7 and CD86 expression on CD103^+^DC. Hence eosinophils may contribute to activation and instructed migration of DCs from the LP to the MLN [88, 89]. Upon eosinophil depletion T_h_2 priming and induction of peanut allergy was abolished in an IL-4 independent fashion [89]. While supporting data were presented, these data need to be further substantiated to strengthen the case for eosinophils playing a key role in the induction of food sensitization.

#### KE 3: Dendritic cell and macrophage migration: translating innate responses into specific T and B cell responses

DCs and macrophages are the major APCs in the intestine, and play both a role in the induction of immune responses. However, only DCs determine the balance between tolerance and sensitization guided by local intestinal co-players e.g. ECs and ILCs. These co-players stimulate the expression of chemokine receptors facilitating sampling of antigens from the lumen (e.g. CX3CR1) and migration (e.g. CCR7, CXCR4) of CD103^+^DC from the epithelial lining or in Peyer’s patches to draining lymph nodes [71].

Intestinal DCs and macrophages, as well as of individual intestinal DC subsets are poorly defined. In the murine intestine, CD103, CD11b, and CX3CR1 expression identifies three major populations of CD11c^+^MHCII^+^ DCs. CD103^+^CD11b^+^CX3CR1^−^ and CD103^+^CD11b^−^CX3CR1^−^ DCs arise from a non-monocytic origin and are considered classical DCs. They express CCR7 and migrate to MLN under steady-state and inflammatory conditions where they affect T cell homeostasis. CD103^−^CD11b^+^CX3CR-1^+/int^ DCs arise from monocytes and resemble macrophages despite lacking F4/80 or CD64 [90, 91]. They do not express CCR7 in the steady state and their ability to migrate to MLN remains controversial. Mice treated with cytokine Fms-related tyrosine kinase 3 ligand (Flt3L), a hematopoietic growth factor, increase the numbers of these DC subsets and a plasmacytoid CD11c^+^B220^+^mPDCA^+^ subset (pDC). The increase correlates with a decrease in IgE response to peanut extract and is reversed by depletion of pDC using e.g. monoclonal antibody 120G8. Thus, pDCs may control food allergic responses and possibly tolerance development [92].

Under basal conditions, CD103^+^CCR7^+^ DCs are known to instruct oral tolerance via the induction of T_reg_ in the MLN. However, CCR7 KO mice lacking CD103^+^MHCII^+^CD86^+^ migratory DCs in the MLN do not develop sensitization and allergy after intragastric exposure to peanut, suggesting this subset may instruct immunity [84, 93–98].

Macrophages may also play a role in food allergic reactions [99], either by exhibiting regulatory or proinflammatory processes [100, 101]. To date comprehensive investigations deciphering the precise role of macrophages in food allergy is lacking.

#### KE 4 and 5: Organ responses: initiation and amplification of specific responses

##### KE 4: T cell priming, proliferation and polarization

**Fig. 3:**
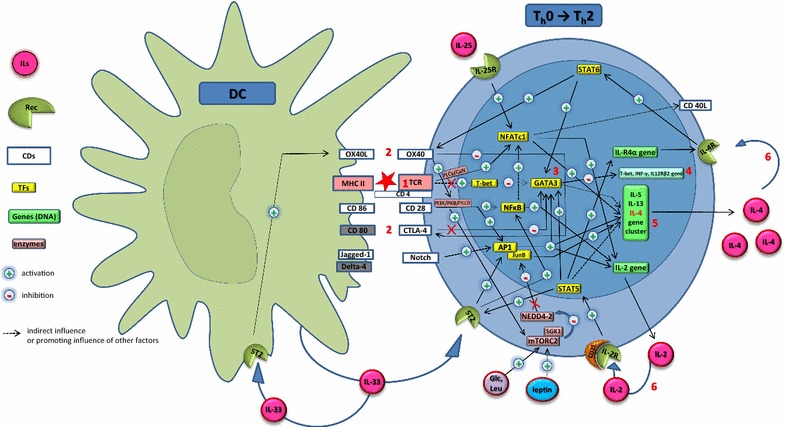
Differentiation of naïve Th cells into Th2 cells. The T cell receptor (TCR) is triggered by the specific recognition of allergenic peptides presented by MHC molecules (MHC II) (event 1). Even in the absence of TCR triggering, the co-stimulatory and co-inhibitory receptors on the T cells direct the T cell polarization and determine the T cell fate (events 2). The upregulation of transcription factor GATA-3 (event 3) is essential in the polarization towards Th2 since it suppresses Th1 and Th17 polarizing events (event 4) while promoting the IL-4, IL-5 and IL-13 gene translation (event 5), the so called keystones of the Th2 response. Cytokines produced by these polarized Th2 cells, including IL-2 and IL-4 can self-amplify the differentiation process (events 6). *ILs* interleukins, *Rec* receptors, *CDs* cluster of differentiation, *TF* transcription factors

Molecular profiling of human T cell pathways indicate that the signal supplied by the T cell receptor (TCR) (Fig. 3, event 1) only participates in the development of the T_h_1 cell phenotype, whereas formation of the T_h_2 phenotype depends on the CD28 signalling, even in the absence of specific TCR activation [102] (Fig. 3, events 2). Stimulation of CD28 activates NF-κB signalling and the expression of GATA3, a signal favouring T_h_2 differentiation [103] (Fig. 3, event 3).

In the process of T_h_1/T_h_2 polarization also CTLA-4 (CD152) appears important. CTLA-4 mediated dephosphorylation of ‘linker for activation of T cells’ (LAT) results in TCR signal inhibition, decreased GATA3 expression, and inhibition of CD28 mediated T_h_2 differentiation [104, 105] (Fig. 3). During sensitization, both CD28 and CTLA-4 molecules may interact with either CD80 or CD86 on the surface of APCs [106, 107] (Fig. 3, events 2). Following exposure to an allergen CD86 expression increases faster than for CD80, suggesting that CD86 is required for initiating the immune response, whereas CD80 has a more regulatory function [106]. Kuchroo et al. [108] showed that CD28–CD86 interaction relates to T_h_2 cell responses, whereas CD28–CD80 interaction favours T_h_1 cells responses. Thus, compounds (e.g. allergens) affecting the kinetics of CD86 and CD80 expression on APCs and CD28 and CTLA-4 expression on T cells, may favour a T_h_2 response.

The abundance of GATA3 in T_h_2 cells (Fig. 3, event 3) triggered the hypothesized that naïve CD4^+^T cells are “naturally” programmed for switching into T_h_2 cells and that T_h_2 cell differentiation is repressed by T-bet during T_h_1 cell differentiation in healthy individuals [109] (Fig. 3, event 4). The importance of GATA3 for the T_h_2 cell phenotype is well established [110]. It promotes the IL-4/IL-5/IL-13 gene translation (Fig. 3, event 5) by regulating methylation of histones which grants access for transcription factors (e.g. c-Maf) that activate IL-4 gene expression. GATA3 also binds specific DNA sites controlling activation (e.g. IL-5 and IL-13) or inhibition (e.g. T-bet, IFN-γ and IL-12Rβ2) of cytokine expression [110, 111].

IL-2 from activated CD4^+^T cell plays a crucial role in the T_h_2 cell polarization [112] (Fig. 3, event 6). Activation of the IL-2 signalling via STAT5 results in an early and IL-4-independent induction of the IL-4Rα subunit with formation of a functional IL-4 type I receptor, the signalling pathway for IL-4 [113]. IL-2/STAT5 may also participate in the early initiation of the IL-4 gene expression [114]. The mechanisms driving the establishment of IL-4 signalling by activated CD4^+^T cells resembles OX40L/OX40 signalling [115]. OX40 (CD134) expression is induced on the surface of naïve T cells hours after exposure to antigen [116]. Allergen-induced T_h_2 polarization requires only endogenous IL-4 and is controlled by the OX40L/OX40 signalling pathway [115]. OX40 signalling sustains TCR, CD28 and IL-2R expression [116].

The contribution of TSLP, IL-25 and IL-33 to T_h_2 polarization is recognized in a murine asthma model. Influence of TSLP on the T_h_2 response development is indirect and occurs by inhibiting IL-12 secretion and by inducing OX40L co-signalling in DCs. The presence of IL-25R and IL-33R on CD4^+^T cells suggests that IL-25 and IL-33 exert a direct effect on these cells. In vitro IL-25 promotes T_h_2 differentiation and IL-4 expression through NFATc1 and IL-4/STAT6-dependent mechanisms [117]. This observation was not confirmed by a murine model for HDM allergy [82]. Epithelial IL-33 affects OX40L expression by DCs and activates signalling pathways that are relevant for the T_h_2 polarization (e.g. ERK, MAPKs and NF-κB) via IL-33R. Abolishing IL-33 signalling in allergic mice decreased the production of specific IgE with 50% [82, 118]. However, whether IL-33 is inducing or maintaining a T_h_2 phenotype cytokine remains to be clarified.

Activation and differentiation of naïve CD4^+^T cells may be under hormonal and metabolic control. A key event for both immune and metabolic signalling is mTOR protein kinase (PK) activity. A HDM/OVA mouse model for asthma showed that mTORC1 activity relates to T_h_1 and T_h_17, while mTORC2 relates to T_h_2 differentiation [119]. A role of mTOR PK, if any, in sensitization seems plausible. Inhibition of mTOR PK prevents activation of STATs and T-bet expression, known to be involved in sensitization, and drives iT_reg_ cell differentiation by blocking polarization signals even in the presence of the T_h_1 and T_h_2 polarization cytokines. A leptin-dependent increase of the mTOR signalling is one of the causes of iT_reg_ cell developmental disturbances resulting in food allergies [119, 120].

##### KE 5: B cell activation and class switching

Synthesis of specific IgE requires two different levels of qualitative changes in the DNA of immunoglobulin (Ig) genes. Somatic hypermutation (SHM) in the variable regions of heavy and light chain leads to changes in the affinity to antigen. Direct or sequential Ig class-switch recombination (CSR) constitutes strictly controlled intrachromosomal DNA fragment deletion (class segments) in the constant region of heavy chain (IGH) locus [121]. In atopic dermatitis patients the CSR for IgE antibodies may occur in a direct way (IgM > IgE) or an indirect one (IgM > IgG > IgE) [122–124].

**Fig. 4:**
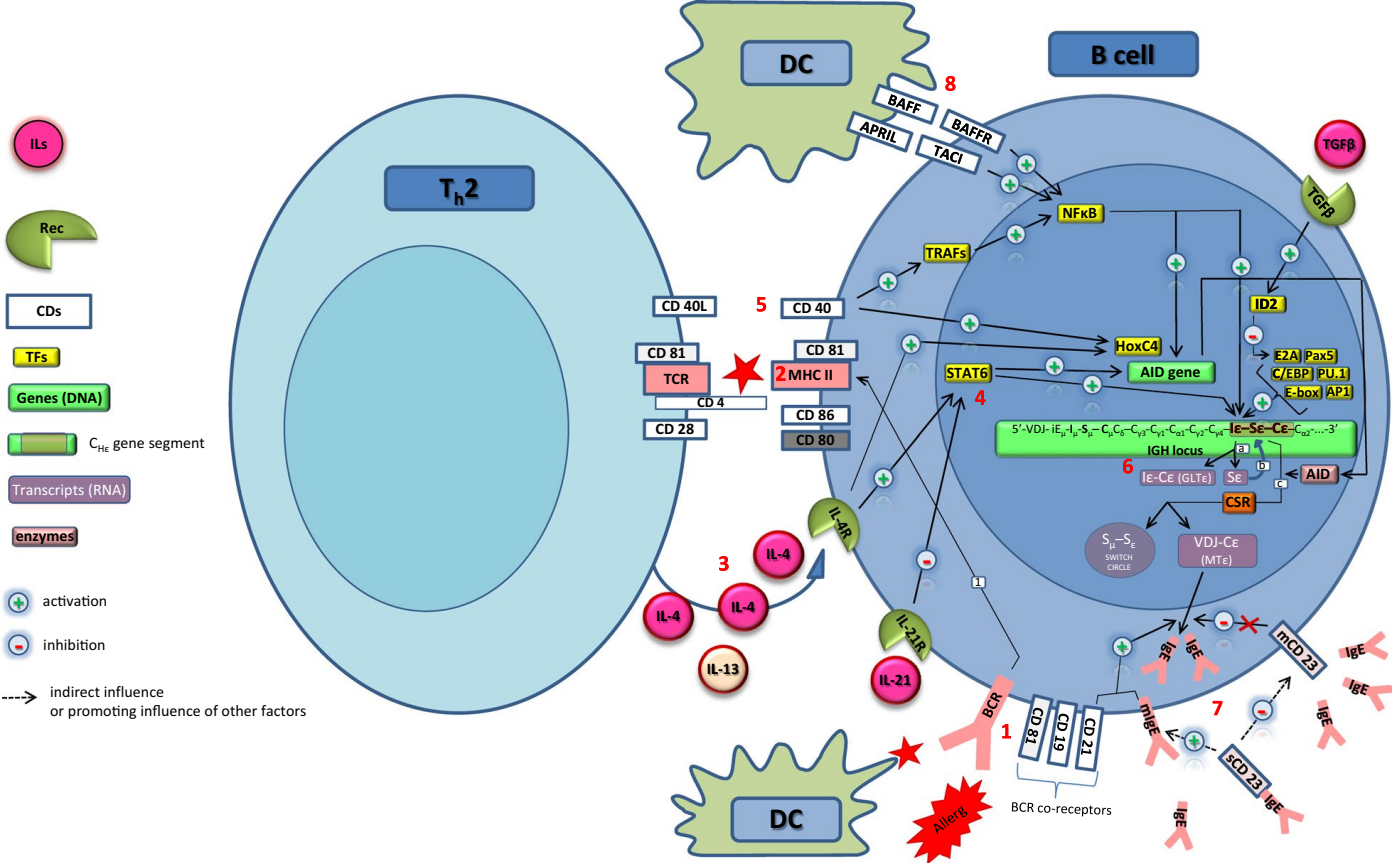
IgE isotype switching in B cells. The surface immunoglobulin that serves as the B-cell antigen receptor (BCR) has two roles in B-cell activation. First, in combination with the BCR co-receptors, it transmits signals directly to the cell’s interior when it binds antigen (event 1). Second, the B-cell antigen receptor delivers the antigen to intracellular sites where it is degraded and returned to the B-cell surface as peptides bound to MHC class II molecules (event 2). Subsequent isotype switching of B cells to IgE production is induced by two separate signals, both of which can be provided by polarized Th2 cells. The first signal is provided by the cytokine IL-4 interacting with the IL4 receptor on the B cells (event 3), which leads to activation of STAT-6 (event 4). The second signal for IgE switching is a co-stimulatory CD40-CD40L interaction (event 5). Both signals are important for the initiation of the antibody class switching (event 6), resulting in IgE production (event 7). It is also possible to induce a CD40-independent induction of isotype switching which involves the interaction of BAFF-BAFFR and APRIL-TACI interactions between DC and B cells (event 8)

The surface B cell antigen-recognizing receptor (BCR) identifies the antigen in its native form. BCR stimulation triggers a complex cascade of signalling events leading to B cell activation [125] (Fig. 4). The CD19/CD81/CD21 complex on B-cells is important for B cell activation with CD81 playing a crucial role in B-cell/T-cell communication through the MHCII-TCR (Fig. 4). Activated B cells establish contact with T_h_2 cells by interaction of the CD80/86–CD28 and CD40–CD40L molecules (Fig. 4).

Effective induction of the activation-induced cytidine deaminase (AID) expression requires a cooperation between the CD40/TRAFs/NFκB signalling pathway, IL-4 and IL-13 as well as CD40 and IL-4 activation of HoxC4 in the B cell nucleus (Fig. 4). CD40 signalling in combination with IL-4 and IL-13 activates the synthesis of GLTɛ. Activation of the Iɛ promoter by transcription factors Pax5, PU.1, C/EBP, AP1, and E-box for E2A enhances GLTɛ transcription. GLTɛ synthesis is regulated by IL-4 only, since IL-4 depletion is sufficient to block Ig CSR to IgE [126–128]. Ig CSR to IgE may occur also in the absence of CD40 signalling [122–124]. BAFF and APRIL molecules on DCs activate surface BAFFR and TACI molecules on B cells, and stimulate NFκB through a pathway involving NIK (NFκB-inducing kinase) and p52 activation (Fig. 4). IL-4-secreting mast cells, eosinophils, basophils, and γδ T cells, are alternative inducers of CD40 independent IgE synthesis [104, 128].

That IgE synthesis is tightly regulated is suggested by the minute amounts of serum IgE as compared to IgG, even in allergy or parasitic diseases. One regulatory mechanism involves rapid capturing and subsequent endocytosis by cells expressing a high affinity receptor FcɛRI (e.g. on mast cells, basophils or eosinophils), and to a lesser extent by the cells expressing a low affinity receptor FcɛRII/CD23 (e.g. intraepithelial cells (IECs) or B cells). Due to technical challenges the mechanistic understanding of IgE synthesis regulation and Ig class switch at cellular level is limited, especially in humans [122, 129]. IgE switching may result in suboptimal polyadenylation of 3′-untranslated region of membrane IgE resulting in less stable mRNA for membrane IgE (mIgE) and lower expression of mIgE BCRs [129]. A plasma membrane molecule specifically involved in the negative feedback regulation of IgE-CSR is CD23 (FcεRII), which plays a role in in vivo sensitization induction by cysteine proteases (e.g. HDM Der p 1) [130]. In the presence of high levels of IgE or IgE-antigen complexes membrane-bound CD23 (mCD23) suppresses the production of IgE. Endogenous or exogenous (e.g. by Der p 1) proteolytic cleavage of mCD23 renders CD23 soluble (sCD23) which reinforces the production of IgE by inhibiting binding of IgE to mCD23. In combination with CD21 mediated signalling, sCD23 enhances B cell proliferation and transformation into IgE + plasma cells. Three cytokines may also be involved in down-regulation of IgE synthesis. IL-21 represses IL-4 stimulated GLTɛ synthesis in a STAT6-independent manner. IFN-γ increases IgG1-CSR over IgE-CSR, whereas TGF-β induces transcriptional factor ID2 (inhibitor of DNA binding-2) that suppresses the E2A binding with Iɛ promoter [131, 132].

### Defining sensitization induction mechanisms by getting a grip on tolerance

The current perception is that soluble antigens are predominantly absorbed by EC and suppress the immune response, while particulate or aggregated antigens are taken up by M cells and activate local or systemic immune responses [39, 40]. Nonetheless, divergent results exist on the contribution of these two main routes of intestinal transport for food proteins and on influence of the protein physico-chemical properties [37, 41]. Linking entry route of food proteins and elicited immune response deserves thus further investigation [133].

Oral tolerance to food proteins is the result of anergy and/or deletion of antigen-responsive T_h_2 cells (high-dose tolerance), and differentiation of regulatory T (T_reg_) cells (low-dose tolerance) [134]. The best characterized T_reg_ cells in terms of induction of oral tolerance are FOXP3^+^ T_reg_. Among human FOXP3^+^ cells, thymus-derived CD25^+^FOXP3^+^ natural T_reg_ (nT_reg_) and peripheral induced CD25_bright_FOXP3^+^CD127^−^ T_reg_ (iT_reg_) exist. Both employ several mechanisms to inhibit the activity of allergen-specific T_h_2 cells and promote the synthesis of allergen-specific IgG4 by B cells [135, 136].

Oral tolerance depends on the ability of CD103^+^ DCs to induce FoxP3^+^α4β7^hi^ CCR9^hi^ iT_reg_ in the MLN. The molecular conditions for tolerance to develop include conversion of vitamin A into retinoic acid (RA), upregulation of TGF-β, enzyme indoleamine 2,3-dioxygenase (IDO), intestinal Muc2, GM-CSF and co-signalling molecule 4-IBB [137–139]. This micro-environment facilitates gut-homing receptors CCR9 and α4β7 mediated migration of primed T_reg_ cells from the MLN to the LP of the small intestine [140]. In the LP T_reg_ cells are expanded and maintained by IL-10 producing CD103^−^CX3CR1^+^ macrophages resulting in oral tolerance development [141, 142]. IL-10 and IL-27 producing CD11b^+^DCs enhance the secretion of IL-10 by T_reg_ cells [143].

Induction of iT_reg_ cells depends on a low density of high-affinity ligands on DCs, sub-optimally activation of TCR signalling, induction of CD28, and lack of the CTLA-4 signalling. These cells synthesize IL-2 and their transient CD25 expression coincides with CD25 expression by T_h_2 cells [144, 145]. Both in vitro and in vivo iT_reg_ cells require the presence of the GATA3 for maintaining FOXP3 expression and accumulation in inflammatory sites. In contrast to T_h_2 cells, GATA3 expression on iT_reg_ cells regulation is IL-4/STAT6-independent but controlled by IL-2/STAT5 [146]. Extracellular adenosine reinforces CD25, CTLA-4 and FOXP3 expression by increasing the cAMP concentration in naïve CD4^+^T cells and, at the same time, preventing ZAP70 phosphorylation, and the AKT and ERK1/2 activation, which inhibits stimulation, differentiation and proliferation of the remaining T cell subsets [147].

Regulatory B cells (B_reg_) with tolerogenic functions exist in human and mice. These B_reg_ also express FoxP3 and produce IL-10, TGF-β and IL-35 [148]. In humans with cow’s milk allergy the frequency of specific IL-10-producing B cells is higher than in healthy individuals [149, 150]. Studies using mouse models of food allergy revealed that allergen-specific CX3CR1^+^ B_reg_ cells producing TGF-β and IL-10 are expanded in response to high-dose allergen exposure [151].

### The currently most substantiated building blocks of the AOP for food sensitization

This proposed AOP used the one outlined for skin sensitization [12] and the AOP suggested for sensitization of the respiratory tract [13] to capture data that are available for proteins in general, and supplemented it with data on sensitizing food and food proteins in particular. Available mechanistic data on protein respiratory sensitization [14] were included to fill out gaps in the understanding of how proteins may affect cells, cell–cell interactions and tissue homeostasis. Therefore, caution should be considered since the skin and the respiratory tract may differ in view of food intake, tolerance, microbiome, presence of digestive enzymes, hormones etc.

Efforts to discern the mechanisms involved in food sensitization are motivated by the need for test methods and strategies to identify novel food with sensitizing potential. An AOP approach allows to transparently gather all available and relevant information following recognized criteria [11]. This assessment subsequently informs potential regulatory applications, which may include support for grouping and read-across of food and food proteins, identification of relevant and biologically plausible test methods, support for the development of integrated approaches to testing and assessment (IATA), identification or characterization of hazard, or quantitative risk assessment.

The potential regulatory applicability of any AOP is informed by the degree of confidence in the biological plausibility of each of the key event relations (KERs), and the identified KEs, and the empirical support for each of the KERs and the overall AOP [11].

#### KER 1: MIE triggers innate responses and inflammation at epithelial level (KE 1)

The MIEs for food sensitization induction by food and food proteins are poorly understood. The knowledge and understanding acquired for proteins sensitizing the respiratory tract provides insight into MIEs potentially triggered by proteins: modification of tight junctions (MIE-1), receptor-mediated effects (MIE-2) (e.g. PAR and TLR signalling) and endocytosis facilitating modification of intracellular processes (MIE-3) [14].

The scarce data on food proteins suggest that, in analogy with respiratory sensitizing proteins, cellular danger signals, induction of oxidative stress and pro-inflammatory cytokines and chemokines are involved in sensitization induction. The proposed MIEs for food proteins include modification of tight junctions (MIE-1) by proteolytic (e.g. kiwi Act d 1) but also non-proteolytic (e.g. gluten gliadin and peanut Ara h 2) allergens, receptor mediated induction of inflammation (MIE-2) (e.g. CD23 mediated) and unspecific endocytosis with impact on intracellular events (MIE-3). The proposed MIE-2 for food proteins involves CD23-mediated uptake by the exposed cells. Whether this event occurs also during sensitization induction by food proteins and not only during elicitation remains to be substantiated. Alternative MIE-2 mechanisms for proteolytic food allergens (e.g. kiwi Act d 1) may exist. CD23 was reported to play a role in sensitization induction in the respiratory tract, but not as a carrier for endocytosis. HDM Der p 1 and Der f 1, both cysteine proteases, release CD23 from the membrane and increase the concentration of sCD23, resulting in a disturbance of the negative control of IgE production [130]. A role of PAR-2 in receptor mediated induction of sensitization by proteolytic allergens is not established yet. However, an analogy with Der p 1 and Der f 1 cannot be excluded [24].

The causal relation between these MIEs and the induction of ROS, T_h_2-driving cytokines (e.g. IL-33, TSLP and IL-25) and eventually inflammation is well established. Especially epithelial IL-33 is a major player both in the induction of sensitization, and the activation of e.g. DCs, basophils, mast cells and eosinophils [152]. IL-33 activates signalling pathways (e.g. ERK, MAPKs, NF-κB) with relevance for inflammation and T_h_2 polarization. TSLP inhibits IL-12 secretion while stimulating OX40L co-signalling on DCs. The presence of IL-25R on CD4^+^ T cells suggests that IL-25 exerts a direct effect on these cells [117].

#### KER 2: MIE 2–3 trigger innate responses and inflammation at DC level (KE 2)

Except MIE-1 (tight-junction modification), the identified MIEs may also have relevance for DCs since these cells share the innate mechanisms (e.g. PRRs, TLRs) reacting with e.g. PAMPs and DAMPs.

Differences between MIE-2 (receptor-mediated) effects on ECs (expressing PAR-1, 2, 3 and 4) and DCs (expressing primarily PAR-1) may exist. In vitro studies using human primary ECs and cell lines revealed that the cysteine protease Der p 1 activates PAR-2 but inactivates PAR-1 [153]. Since PAR-1 is the dominant PAR on the surface of DCs, it may be anticipated that DC activation is reduced by allergens that engage PAR-1. While it remains to be substantiated mechanistically, the available data suggest that e.g. HDM may directly suppress T_h_1 driving DC responses [82].

In vitro and in vivo data underscore the importance of IL-33 for proper activation of DCs as determined by e.g. increased OX40L expression. Allergenic proteins in the presence of IL-33 drive activated DCs to release IL-1β, IL-6 and IL-10, with IL-1β known to be involved in T_h_2 cell stimulation and antibody production [82, 86].

#### KER 3: KE 1–2 drive DC migration (KE 3)

The ECs (IL-33, TSLP, IL-25), OX40L^+^ DCs (IL-1β, IL-6 and IL-10) and eosinophils (EPO) derived signals constitute an allergen-induced inflammatory microenvironment that triggers DCs maturation and migration.

Among the currently identified DC subsets, CD103^+^CD11b^−^CX3CR1^−^ and CD103^+^CD11b^+^CX3CR1^−^ DCs appear to be the most relevant for sensitization induction. Informed by changes in the microenvironment at epithelial level, CD103^+^ DCs express chemokine receptors facilitating sampling of protein from the lumen (e.g. CX3CR1), MHC II, co-stimulatory molecules (e.g. CD54, CD80, CD86 and OX40L)) as well as receptors that are required for migration to the LP and MNL (e.g. CCR7 and CXCR4).

Especially CD103^+^MHCII^+^CD86^+^ DCs expressing CCR7 seem to be important for sensitization induction.

#### KER 4: KE 3 leads to T and B cell activation in the lymphoid organs (KE 4–5)

It is well documented that epithelial TSLP, IL-25 and IL-33 play an important role also in T_h_2 polarization, by inhibiting IL-12 secretion while stimulating OX40L co-signalling on DCs (TSLP) and IL-33R mediated activation of DCs and CD4^+^ T cells (IL-33). The mechanism by which IL-25 contributes to T_h_2 polarization is not clear, but the presence of IL-25R on CD4^+^ T cells suggests that IL-25 exerts a direct effect on these cells [117] [82, 118].

Polarization of the T cell response towards T_h_2 requires GATA3 which promotes the expression of IL-4, IL-5 and IL-13 while inhibiting T-bet, IFN-γ and IL-12Rβ2 [110, 111]. Furthermore, activation of autocrine IL-2 signalling drives the establishment of the IL-4 signalling pathway by an IL-4 independent induction of the IL-4R subunit and formation of a functional IL-4 type I receptor [113].

Proper T_h_2 activation requires the expression by T cells of CD28, OX40 (CD134) and CTLA-4 (CD152) and binding of these marker proteins to CD80, CD86 and OX40L on the surface of the activated DCs [104, 105]. In this context, CD86 seems to be most relevant for induction of sensitization [106]. Indeed, CD28–CD86 interaction relates to T_h_2 cell responses, whereas CD28–CD80 interaction favours T_h_1 cells responses [108]. It is suggested that the allergenic potential of proteins is defined by their capacity to affect the kinetics of the CD86/CD80 expression on DCs and CD28/CTLA-4 expression on T cells, in the context of MHCII-peptide-TCR interaction.

The ultimate marker for sensitization to protein is the production of specific IgE. The CD19/CD81/CD21 complex on B-cells is important CD81 playing a crucial role in B-cell/T-cell communication through the MHCII-TCR. Interaction between T_h_2 cells and activated B cells requires functional CD86–CD28 and CD40–CD40L interactions [125].

IL-4 appears to be sufficient for Ig CSR to IgE to occur. This cytokine may originate from activated T_h_2 cells (CD40 dependent) or mast cells, eosinophils, basophils and γδ T cells (CD40 independent). CD40 signalling in combination with IL-4 and IL-13 activates the synthesis of GLTɛ [126–128]. CD40 independent mechanisms involve the interaction between BAFF and APRIL on DCs, and BAFFR and TACI on the surface of B cells [122–124]. This interaction stimulates NFκB signalling through a pathway involving NIK (NFκB-inducing kinase) and p52 activation [104, 128].

#### Tolerance

Developing AOPs for sensitization induction by foods and food proteins requires sufficient understanding of the mechanisms behind tolerance development.

Overall, tolerance induction seems to be the result of CD103^+^ DCs mediated stimulation of FoxP3^+^α4β7^hi^ CCR9^hi^ iT_reg_ in the context of complex RA-rich environment in the MLN. Establishment of iT_reg_ in the LP requires IL-10 from CD103^−^CX3CR1^+^ macrophages.

Despite iT_reg_ cells believed to be involved in tolerance development, food antigen-specific iT_reg_ cells have been detected in both allergic patients and healthy people. It is hypothesized that the equilibrium between allergen-specific T_h_2 cells and iT_reg_ cells, which recognize the same epitope, predisposes to an allergic response (T_h_2) or a healthy tolerogenic response [154].

In contrast to iT_reg_, nT_reg_ cells constitutively express CTLA-4 and are believed to limit the access to and activation of naïve CD4^+^ T cells by aggregation with DCs, by IL-10 and TGF-β mediated inhibition of MHC-II expression on DCs, and effector functions and migration of activated T and B cells, while promoting iT_reg_ cell differentiation [154].

Although the mechanisms underlying these effects remain unclear, the identification of new B_reg_ subsets and functions encourages intensifying the research into their share in tolerization versus sensitization to food proteins.

## Conclusion

This manuscript has collected, structured and evaluated molecular and cellular information on protein sensitization in general, and food sensitization in particular with the aim to build AOPs for food sensitization. Analysis revealed several KEs and biomarkers (Fig. 1) that may have potential use in testing and assessment of proteins for their sensitizing potential. In the future, this may help to identify a number of methods, each addressing a specific KE, that provide information about the food allergenic potential of new proteins. When applied in the context of an integrated strategy these methods may reduce, if not replace, current animal testing approaches.

The proposed AOPs will be shared at the www.aopwiki.org platform to expand the mechanistic data, improve the confidence in each of the proposed KEs and KERs, and allow for the identification of new, or refinement of established, KEs and KERs.

## Abbreviations

Ag: antigen
AOP: adverse outcome pathway
B_reg_: regulatory B cells
CLRs: c-type lectin receptors
CSR: class-switch recombination
DAMPs: danger-associated molecular patterns
DC: dendritic cell
EC: epithelial cell
GALT: gut-associated lymphoid tissue
GI: gastro-intestinal
HA: hyaluronic acid
HDM: house dust mite
HWP: hydrolysed wheat proteins
IEL: intraepithelial lymphocytes
IgE: immunoglobulin E
IL: interleukin
ILC: innate lymphoid cells
KE: key event
KER: key event relation
KO: knock out
LP: lamina propria
MC: mast cells
Mϕ: macrophages
MIE: molecular initiating event
MLN: mesenteric lymph node
MOA: mode of action
MW: molecular weight
NKT: natural killer cells
OECD: Organisation for Economic Cooperation and Development
OVA: ovalbumin
PAMPs: pathogen-associated molecular patterns
pDC: plasmacytoid dendritic cell
PRR: pattern recognition receptor
ROS: radical oxygen species
TCR: T cell receptor
TLR: toll-like receptor
T_reg_: regulatory T cells
